# Body image dissatisfaction in patients with inflammatory bowel disease: a systematic review protocol

**DOI:** 10.1186/s13643-018-0844-0

**Published:** 2018-11-13

**Authors:** Sophie Elizabeth Beese, Isobel Marion Harris, David Moore, Janine Dretzke

**Affiliations:** Institute of Applied Health Research, Public Health Building, University of Birmingham, Birmingham, B15 2TT UK

**Keywords:** Systematic review, Inflammatory bowel disease, Ulcerative colitis, Crohn’s disease, Body image

## Abstract

**Background:**

Inflammatory bowel disease (IBD) is a debilitating chronic disease characterised by inflammation and ulceration of the gastrointestinal tract. It is associated with a range of debilitating symptoms and reduced quality of life. People living with IBD may also be at risk of body image dissatisfaction (BID). BID is a distorted and negative view of the physical self, which in turn can adversely affect mental health and quality of life. To date, there have been no systematic reviews of the evidence on BID in IBD patients. Therefore, the aim of this systematic review is to clarify the evidence base on BID in *IBD patients including (i) the tools used to measure BID, (ii) the prevalence and severity of BID, (iii) the risk factors associated with BID and (iv) the relationship between BID and quality of life.

**Methods:**

Bibliographic databases (EMBASE, MEDLINE, PsycINFO, Cochrane CENTRAL) will be searched using a sensitive search strategy aiming to identify any quantitative study reporting on body image in the context of IBD. This will be supplemented by searches of ongoing trials registers and checking of reference lists. Studies will be assessed for eligibility using predetermined selection criteria for each question. Data will be extracted using a predefined data extraction form, and risk of bias (quality) of included studies will be assessed based on checklists appropriate to the study designs identified. Key methodological steps will be undertaken in duplicate to minimise bias and error. Synthesis will be undertaken separately for the different systematic review sub-questions. Given the anticipated heterogeneity of evidence on each question, it is likely that synthesis will be mostly narrative.

**Discussion:**

To the best of our knowledge, this will be the first systematic review to collate the existing evidence on BID in IBD patients. Understanding the impact of BID, its relationship with quality of life, and which patients may be at greater risk, may ultimately lead to the development of interventions to prevent or treat BID and to better patient care. Any gaps in the identified evidence will help to inform the research agenda in this area.

**Systematic review registration:**

PROSPERO: (CRD42018060999).

## Background

Inflammatory bowel disease (IBD) is a chronic gastrointestinal disease characterised by inflammation and ulceration of the gastrointestinal tract. IBD consists of two main forms: Crohn’s disease and ulcerative colitis [1]. Ulcerative colitis affects the large intestine, whereas Crohn’s disease can affect the entire digestive tract [2]. Around 300,000 people in the UK are affected by Crohn’s or colitis [3], as well as 2.5 million across Europe and over 1 million in the USA [4]. IBD is often diagnosed at a young age and equally in men and women. Symptoms are often debilitating and can affect day-to-day activities [5]. Patients may suffer from severe abdominal pain, bloody diarrhoea, weight loss, fatigue and other symptoms such as anaemia, vomiting and fever [6, 7]. An individual may have periods of time when their illness flares up, as well as times when they are in remission [2].

Currently, the cause of IBD is unknown; however, genetic and environmental factors are thought to play a part and IBD is considered as an autoimmune disease [8, 9]. Although there is no cure, treatments include aminosalicylates and steroids to reduce inflammation and immunosuppressants to reduce the autoimmune response in the gastrointestinal tract [10, 11]. Around 20% of patients do not respond to treatments and surgery is considered as an option to remove or repair the damaged part of the digestive system [2]. Studies have also shown that patients with IBD may have a reduced health-related quality of life compared to the general population, particularly when their disease is active and more so in women than in men [12, 13].

Body image is how an individual perceives themselves physically and body image dissatisfaction (BID) is associated with a distorted and negative view of oneself causing feelings of anxiousness and discomfort [14]. Generally, women are more likely to suffer with BID than men [15]. Furthermore, having negative body image can have a serious impact on health and well-being [16]. Studies have shown that patients with negative body image are more likely to suffer with depression, anxiety and suicidal feelings [17]. BID can also impact negatively upon relationships [18] and quality of life [19].

Society has an impact on an individual’s perception of body image with increased pressure on both men and women to adhere to “ideal” bodies and consequentially some people are more critical of themselves [20]. Aspects such as body weight, body shape and skin problems such as acne, may contribute toward an individual’s negative body image [21].

People who live with IBD may be at risk of BID for various reasons. Condition-specific symptoms like swelling of the stomach, anaemia and the less common erythema nodosum (painful red skin nodules) may be an issue [2, 22]. Factors such as disease activity, severity and fatigue may also contribute to the dissatisfaction with body image experienced by the patient. Therapies also have an impact and steroid use is often associated with weight gain, acne, mood swings and “moon face” [23] (when fat is redistributed to the sides of the face and excess water retention occurs [24]). Surgical resection of the bowel causing cosmetic scarring and the implementation of a stoma may also add to BID [25, 26].

Most IBD patients are diagnosed at adolescence [2], a time in life when body image becomes more important and self-consciousness rises. Adolescents are at risk of mental health issues like anxiety and depression [27] and this risk is increased by long-term physical illnesses like IBD [28]. This could be further intensified by BID, leading to possible extreme reactions such as self-harm or feeling suicidal [29, 30].

Body image is not currently considered as an important aspect of care and treatment for IBD patients, although some research suggests that it could potentially be a problem [22]. As body image has been highlighted as an issue in other conditions such as cancer and in transplant patients [31], it is possible it may impact on IBD patients too. Studies in these areas [32, 33] have shown that body image may be associated with reduced quality of life measures.

## Current evidence and rationale

Scoping searches for existing evidence were carried out in EMBASE, MEDLINE, the Cochrane Library and PROSPERO (November 2017) using various terms to encompass IBD and body image (including *inflammatory bowel disease*, *ulcerative colitis*, *Crohn*’*s disease*, *IBD*, *body image*, *body dissatisfaction*). No existing or ongoing systematic reviews on body image in IBD were identified. However, the searches identified in excess of 20 primary studies relating to different aspects of body image in IBD. Studies variously explored measurement tools for BID, prevalence, factors associated with BID in IBD and quality-of-life. A systematic review is therefore warranted to synthesise and clarify this heterogeneous evidence base.

The following four questions will be addressed in this systematic review in order to develop an understanding of the evidence around BID in IBD patients:

1. What tools are used to measure body image in IBD patients and what are their components?
2. What is the prevalence and severity of body image dissatisfaction in IBD patients?
3. What factors are associated with body image dissatisfaction in IBD patients?
4. Is there an association between body image dissatisfaction and quality of life in IBD patients?

## Methods

This systematic review protocol has been prepared according to the Preferred Reporting Items of Systematic Reviews and Meta-Analysis Protocols (PRISMA-P) [34] statement and is also registered on PROSPERO (CRD42018060999).

### Search strategy

Bibliographic databases (EMBASE, MEDLINE, PsycINFO, Cochrane CENTRAL) will be searched using a sensitive search strategy aiming to identify any study reporting on body image in the context of IBD. Index and free text terms for IBD and body image will be combined. Strategies will be adapted for each database and run without date or language restrictions. No study design filters will be applied. Ongoing trials databases (Clinicaltrials.gov and EU Clinical Trial Register) will be searched for any current trials assessing body image. Reference lists for each article included in the systematic review will also be checked. Results will be managed using Endnote X7 (Clarivate Analytics) to facilitate automatic and manual removal of duplicate records, study screening and selection and record keeping. An example search strategy for MEDLINE is shown in Table 1.

**Table 1:** Example search strategy for MEDLINE database

Search	Query
#1	exp inflammatory bowel diseases/
#2	inflammatory bowel disease*.mp.
#3	exp Colitis, Ulcerative/
#4	ulcerative colitis.mp.
#5	exp Crohn disease/
#6	Crohn* disease.mp.
#7	Crohn*.mp.
#8	IBD.mp.
#9	CD.mp.
#10	UC.mp.
#11	1 OR 2 OR 3 OR 4 OR 5 OR 6 OR 7 OR 8 OR 9 OR 10
#12	exp body image/
#13	body image.mp.
#14	body dissatisfaction.mp.
#15	body awareness.mp.
#16	body concern*.mp.
#17	body attitude*.mp.
#18	body preoccupation.mp.
#19	body perception.mp.
#20	body anxiety.mp.
#21	body conscious*.mp.
#22	12 OR 13 OR 14 OR 15 OR 16 OR 17 OR 18 OR 19 OR 20 OR 21
#23	11 AND 22

### Screening and selection criteria

All identified records will be screened by two reviewers (SB and IH) independently based on the criteria listed below. Full texts will be acquired for any potentially relevant studies. Where articles cannot be obtained, authors will be contacted. Reasons for exclusion of studies will be recorded. Should potentially relevant non-English language studies be identified, part translation of relevant portions of articles will be undertaken where feasible. Selection criteria are as follows:

- Question 1. What tools are used to measure body image in IBD patients and what are their components?

Study design: any primary studies reporting quantitative data will be eligible. The most likely study designs will be cross-sectional and cohort studies, but it is possible that relevant data may be reported as part of a randomised controlled trial. Qualitative research will be excluded.

Publication type: conference abstracts identified in searches of bibliographic databases will be included if they were published up to 3 years before date of searches and no subsequent full publication can be identified.

Population: patients of any age diagnosed with IBD. At least 50% of population must have IBD unless results are reported separately for sub-groups of individuals with IBD.

Tools: any tool measuring any aspect of body image (including relevant domains or questions of quality of life tools). Tools specific to certain subgroups, e.g. stoma-based questionnaires will only be included if relevant questions on body image are part of the tool.

The following eligibility criteria will additionally need to be met for studies to be relevant for each of questions 2–4:

- Question 2. What is the prevalence of body image dissatisfaction in IBD patients?

Include: studies that report prevalence/frequency and severity of BID (including mean/median body image scores) in IBD patients.

- Question 3. What factors are associated with body image dissatisfaction in IBD patients?

Include: studies where data is reported on associations between any factor in inflammatory bowel disease patients and BID. This might include factors such as age, gender, disease activity, medication, age at onset and disease subtype.

- Question 4. Is there an association between body image dissatisfaction and quality of life in IBD patients?

Include: studies that have looked at the association between results from body image tools and quality of life tools in patients with a diagnosis of inflammatory bowel disease. This might include an association between two separate domains of the same tool (e.g. body image and physical quality of life).

### Data extraction

Data extraction will be performed independently by two reviewers, SB and IH, with discrepancies resolved through discussion or referral to a third reviewer (DM). A pre-designed piloted data extraction sheet will be used. Authors will be contacted if any clarification of reported information is required. Data to be extracted will include study characteristics, patient characteristics and data needed for analysis, with examples shown below.

#### Study characteristics

Study characteristics include study design, inclusion/exclusion criteria for study, recruitment methods, aim of study, follow-up period and study setting.

#### Participant characteristics

Participant characteristics include number of patients, age, gender, type of inflammatory bowel disease, severity of disease, disease activity, BMI, comorbidities and therapy/surgery.

#### Data for synthesis/analysis

Data for synthesis include body image measurement tool, components of body image tools/scales, data on body image dissatisfaction (e.g. body image scores, prevalence, thresholds for determining BID), factors associated with body image dissatisfaction and how this has been quantified, quality of life measures and association between body image and quality of life.

### Quality assessment

Assessment of study quality will be performed alongside data extraction. SB and IH will independently undertake quality assessment, with discrepancies resolved through discussion or referral to DM. Quality assessment will be based on checklists from the Joanna Briggs Institute critical appraisal tools [35]. The most relevant checklists will be those for cross-sectional analytical studies, cross-sectional prevalence studies and cohort studies. Other study designs may report relevant data; for example, relevant cross-sectional data may be presented in the context of a randomised controlled trial. In this case, the quality items for cross-sectional studies will still be relevant.

Important quality items will likely relate, for example, to sample selection, response rate during enrolment in the study and measurement of outcomes in a valid and reliable way.

### Synthesis/analysis

Evidence for each of the four questions will be synthesised separately. Summary tables of study characteristics will be produced for each question as well as an overall risk of bias table. Given the anticipated heterogeneity (e.g. differences in study design, measurement tools, population and outcome statistics), it is likely that synthesis will be narrative, with key findings tabulated. The feasibility of meta-analysis will be considered but will be dependent on clinical and methodological homogeneity. If feasible, a random effects model is more likely to be appropriate.

Relevant outcome metrics that would be considered for pooling are BID prevalence proportions or mean body image scores, odds ratios/risk ratios or correlation coefficients relating to the association between different factors. If meta-analysis is carried out, the *I*^2^ statistic will be used to measure the percentage of variation across studies that is due to heterogeneity beyond that expected by chance. If meta-analysis is not feasible, it may still be possible to present findings in forest plots (without pooling) for illustrative purposes.

Important subgroups relate to disease type and severity, body image measurement tool, gender and age. Where possible, findings will be presented according to different diseases/disease severities and different tools. Further consideration of subgroups will likely be limited to those reported within primary studies. Quality assessment findings will be used in considering the strength of evidence for each of the four questions. Formal sensitivity analysis based on quality assessment findings is unlikely to be feasible. Funnel plots will be used to assess publication bias alongside Egger’s test [36] for each outcome were meta-analysis is possible and where there are ten or more studies contributing data.

## Discussion

To the best of our knowledge, this will be the first systematic review exploring BID in IBD patients. It will reveal all evidence on BID in IBD including how it is measured, the prevalence and severity of BID, possible risk factors in IBD patients and any associations between BID and quality of life. Any gaps in the evidence base will also be highlighted, which will be useful for informing future research recommendations.

Whilst NICE quality standards for IBD recognise enhancing quality of life in their outcome framework [37], neither this nor any other guidelines currently consider body image factors in the treatment and care decision-making in IBD. At present, it is unclear to what extent BID issues impact on quality of life and which, if any, quality of life measures are capturing aspects relating to BID. Elucidating the relationship between body image dissatisfaction and quality of life in this patient group is therefore important.

It is known that individuals with BID are at risk of depression, anxiety and potentially even self-harm [38]. It is also known that individuals are more likely to suffer with mental health issues if they have a long-term physical health condition [39]. If BID is prevalent in IBD patients, they could be at increased risk of these conditions. Therefore, it is important that IBD clinicians and patients are made aware of any risks, which would allow for early intervention to prevent or ameliorate BID. If certain risk factors are found to be associated with BID in IBD patients, these could be used to identify which individuals are particularly at risk.

Ultimately, this might lead to interventions being developed and implemented that aim to improve body image, thereby reducing further mental health risks and improving quality of life.

## Abbreviations

BID: Body image dissatisfaction
IBD: Inflammatory bowel disease
PRISMA: Preferred Reporting Items for Systematic Reviews and Meta-Analyses
PRISMA-P: Preferred Reporting Items for Systematic Review and Meta-Analysis-Protocols
